# Quantum Negotiation Games: Toward Ethical Equilibria

**DOI:** 10.3390/e28010051

**Published:** 2025-12-31

**Authors:** Remigiusz Smoliński, Piotr Frąckiewicz, Krzysztof Grzanka, Marek Szopa

**Affiliations:** 1 Center for International Negotiation, HHL Leipzig Graduate School of Management in Leipzig, 04109 Leipzig, Germany; remigiusz.smolinski@hhl.de; 2 Institute of Exact and Technical Sciences, Pomeranian University in Słupsk, 76-200 Słupsk, Poland; piotr.frackiewicz@upsl.edu.pl; 3 Department of Operations Research, University of Economics in Katowice, 40-287 Katowice, Poland; marek.szopa@uekat.pl

**Keywords:** ethics, negotiation games, quantum game theory, nash equilibrium, cooperation, competition, fairness, self-interest, equity, honesty

## Abstract

This paper applies quantum game theory to three ethical dilemmas that frequently arise in negotiation: cooperation versus competition, self-interest versus equity, and honesty versus deception. Using quantum extensions of selected games such as the Prisoner’s Dilemma, the Ultimatum Game, the Battle of the Sexes, and the Buyer–Seller Game, we examine whether quantization can generate equilibria that improve classical outcomes while also aligning more closely with ethical principles such as fairness, cooperation, and honesty. The analysis shows that quantum strategies, through entanglement and superposition, can sustain cooperative, fair, or honest behaviour as stable equilibria, outcomes that are typically unstable or unattainable in classical settings. The specific outcomes depend on the chosen quantization method, but across cases, the analysis consistently shows that quantum formulations expand the range of solutions in which efficiency and ethical principles co-exist.

## 1. Introduction

Negotiation often involves a persistent tension between maximizing personal gains and adhering to ethical standards. Research on bargaining styles [1,2], fairness norms [3], and information-sharing dilemmas [4] shows that negotiators frequently struggle to balance competitive advantage with ethical responsibility.

Among the many ethical challenges in negotiation, three dilemmas are especially prominent. The first is cooperation versus competition: whether to pursue joint gains or prioritize individual advantage [1,2,5,6]. The second is self-interest versus equity: whether to accept a less favorable personal outcome to achieve a fairer distribution of resources [3,7,8]. The third is honesty versus deception: whether to disclose information truthfully or misrepresent it to gain a strategic advantage [4,9,10].

Classical game theory has persistently returned to these dilemmas, modeling them as recurring features of strategic interaction [11,12,13]. Games such as the Prisoner’s Dilemma, the Ultimatum Game, and the Battle of the Sexes reveal equilibrium strategies that are stable but ethically neutral. Nash equilibria resolve tensions by privileging self-interest, often at the expense of fairness, cooperation, or honesty.

Quantum game theory offers a new perspective. Drawing on principles such as superposition, entanglement, and quantum measurement, it extends the range of possible strategies beyond classical pure or mixed approaches [14,15]. In some cases, quantization enables outcomes that are both more cooperative and more equitable without external enforcement.

Recent research in quantum game theory has expanded well beyond static two-player games, addressing multi-qubit settings, time-dependent quantum games, and nonlinear or chaotic dynamics in economic competition models. Examples include studies on entanglement-driven cellular automata, time-dependent extensions of the Eisert–Wilkens–Lewenstein framework, multi-qubit nonlocal games, and quantum oligopoly models exhibiting nonlinear and chaotic behavior [16,17,18,19,20,21]. While these contributions focus on dynamics, complexity, or physical implementation, the present work addresses a complementary question: how carefully constrained quantum extensions reshape equilibrium selection in canonical negotiation games.

In this study, ethics is not approached as a comprehensive moral or philosophical doctrine, but rather in an operational, game-theoretic sense that is standard in negotiation research and welfare economics. Working within the von Neumann–Morgenstern expected utility framework, we do not alter players’ preferences or impose ethical constraints exogenously. Instead, ethical relevance is evaluated through equilibrium outcome properties that are widely regarded as normatively significant in strategic interactions, such as the ability to sustain cooperation in social dilemmas, to reduce distributive asymmetries in coordination and bargaining games, and to discourage deceptive behavior under incomplete information. Quantum strategies modify the structure of feasible outcome lotteries without changing the underlying utility functions, thereby enabling Nash equilibria with higher expected utilities that simultaneously exhibit these normatively desirable characteristics. In this sense, ethical improvement is understood as an endogenous consequence of an enriched strategic structure rather than as a departure from rational choice theory.

This development raises a timely question for negotiation research: Can the quantization of negotiation games enable the emergence of Nash equilibria that are both strategically stable and consistent with ethical principles such as cooperation, fairness, and honesty?

## 2. Literature Review

Strategic interactions have long been a subject of game-theoretic analysis, often revealing a tension between rational self-interest and cooperative or ethical outcomes. A foundational example is the Prisoner’s Dilemma, in which two players must choose independently between cooperation and defection [22]. While mutual cooperation produces a better collective result, each player has a dominant strategy to defect, anticipating that the other might do the same. The resulting Nash equilibrium, in which both players defect, is stable in the game-theoretic sense but socially suboptimal. Introduced by John Nash [11], the equilibrium concept captures situations in which no player can improve their payoff by deviating unilaterally, yet it remains indifferent to whether the outcome is efficient or fair. This disconnect is particularly salient in negotiation contexts, where outcomes are evaluated not only by their strategic soundness but also by their ethical and distributive implications.

Ethical concerns become especially clear when equilibrium outcomes, though stable, appear normatively undesirable. In the ultimatum game, the Nash equilibrium predicts that proposers will offer the smallest possible positive amount, and responders will accept it. However, Güth et al. [23] demonstrated experimentally that proposers tend to offer more than the minimum, and responders frequently reject low offers, sacrificing personal gain to punish perceived unfairness. These findings have been replicated across cultures [24], showing that fairness preferences systematically shape bargaining behavior. Similarly, Fehr and Schmidt’s [7] model of inequity aversion formalizes how social preferences influence outcomes beyond narrow self-interest.

This evidence has fueled a broader critique: game-theoretic rationality, especially when applied to negotiations, often predicts strategies that are stable or even efficient but ethically problematic. Schelling [25] highlighted the ethical implications of threats and manipulation in strategic bargaining, while Binmore et al. [26] showed that equilibrium predictions frequently diverge from fairness-based judgments. Subsequent studies [8,27,28] reinforced the point that rational strategies in social dilemmas often fail to align with ethical expectations. Negotiation research consistently shows that dilemmas of honesty, trust, and fairness are central to bargaining [4,29,30]. Collectively, these studies emphasize that the stability of Nash equilibria often comes into tension with ethical norms.

In light of these limitations, quantum game theory emerged in the late 1990s as an extension of classical game theory into the domain of quantum mechanics. Eisert et al. [14] pioneered this approach by introducing entangled quantum strategies into the Prisoner’s Dilemma, showing that mutual defection can be avoided when players share entanglement and access a wider set of unitary operations. The dilemma is effectively resolved, yielding higher payoffs for both players than the classical equilibrium allows. This approach was quickly generalized to other games, including the Battle of the Sexes [15] and the Ultimatum Game [31]. In the quantum Battle of the Sexes, entanglement has been shown to symmetrize payoffs and yield fairer outcomes, while quantum ultimatum games allow for responders to employ probabilistic or superposed strategies that reduce the acceptance of unfair offers [31].

Over time, a variety of quantization methods have been developed, differing in their use of initial states, entanglement parameters, strategy spaces, and measurement protocols. These methodological variations fundamentally shape the structure of the resulting game and the equilibria that emerge [32,33,34,35]. Consequently, the capacity of quantum strategies to resolve classical dilemmas depends not only on the underlying game but also on the particular method used to construct its quantum extension.

Despite the growing interest in quantum game theory, research on its ethical implications remains limited. While behavioral game theory has advanced the understanding of fairness, reciprocity, and trust in negotiation [7,8,24], quantum approaches have focused largely on efficiency improvements and Pareto gains. Some work has touched on fairness and regret in quantum ultimatum games [36,37], yet systematic engagement with ethical issues such as cooperation, equity, and honesty remains underdeveloped.

This paper contributes to this emerging area by examining whether quantum game theory can address three central ethical dilemmas in negotiation games: cooperation versus competition, self-interest versus equity, and honesty versus deception. We ask whether the application of quantum strategies to these dilemmas can yield solutions that not only surpass classical outcomes in efficiency but also align more closely with ethical standards of fairness, equity, and transparency.

## 3. Quantum Concepts for Negotiation Games

The following section provides an outline of the basic quantum concepts relevant to this paper. Its purpose is to convey a conceptual overview of qubits, quantum operations, and entanglement.

### 3.1. Qubits, Superposition and Measurement

In quantum information theory, the basic unit of information is the qubit. A qubit is the quantum analog of a classical bit, but instead of being strictly in the state 0 or 1, it can exist in a combination of both. Mathematically, a qubit is a vector with two complex components whose squared magnitudes add up to one (Definition 1). These components, called probability amplitudes, determine the probability of measuring the qubit in one of its two possible basis states, denoted | 0 ⟩ and | 1 ⟩.

**Definition** **1.** *A qubit is represented by a column vector $v=\left(\begin{array}{c} \alpha_{0}\\ \alpha_{1} \end{array}\right)$, where*

- *$\alpha_{0},\alpha_{1}\in C$,*
- *$|\alpha_{0}{|}^{2}+{\left|\alpha_{1}\right|}^{2}=1$.*

Assuming Dirac notation $|0\rangle =\left(\begin{array}{c} 1\\ 0 \end{array}\right)$, $|1\rangle =\left(\begin{array}{c} 0\\ 1 \end{array}\right)$, a qubit can be expressed as a superposition of these basis states $v=\alpha_{0}|0\rangle +\alpha_{1}|1\rangle$. As a result of measuring a qubit *v*, classical information (a bit) is extracted. The probability of obtaining the values 0 and 1 is the square of the modulus of the corresponding amplitude of the quantum state, i.e.,

(1) $$P\left(\mathrm{outcome}\;\mathrm{is}\;i\right)=|\alpha_{i}{|}^{2},\;i=0,1.$$

The very nature of a qubit does not clearly indicate what the essential difference is between a qubit and, for example, a random bit. If we consider a qubit in an equal superposition state $\frac{1}{\sqrt{2}}|0\rangle +\frac{1}{\sqrt{2}}|1\rangle$, then

(2) $$P\left(\mathrm{outcome}\;\mathrm{is}\;0\right)=P\left(\mathrm{outcome}\;\mathrm{is}\;1\right)=\frac{1}{2}.$$

We obtain exactly the same probability distribution when considering a classical random bit with a uniform distribution. The advantage becomes clearly noticeable when we include the set of available operations on the quantum state in our considerations. Operations on a quantum state are represented by unitary matrices.

### 3.2. Quantum Operations and Reversibility

Operations on qubits are described by unitary transformations (Definition 2), which are mathematical operations that preserve probability and are reversible.

**Definition** **2.** *A square matrix U with complex entries is called unitary if it satisfies the condition*

(3) $$UU^{†}=U^{†}U=1$$

*where 1 denotes the identity matrix, and $U^{†}$ refers to the conjugate transpose of U–that is, the matrix formed by first taking the transpose of U and then applying complex conjugation to each of its elements,*

(4) $$U^{†}=\bar{U^{T}}.$$

One particularly important example is the Hadamard gate (5), which transforms a definite state like |0〉 into an equal superposition of |0〉 and |1〉, and |1〉 into a similar superposition with one amplitude of the opposite sign (6).

(5) $$H=\left(\begin{array}{cc} \frac{1}{\sqrt{2}} & \frac{1}{\sqrt{2}}\\ \frac{1}{\sqrt{2}} & -\frac{1}{\sqrt{2}} \end{array}\right).$$

(6) $$H|0\rangle =\frac{1}{\sqrt{2}}|0\rangle +\frac{1}{\sqrt{2}}|1\rangle =|+\rangle ,\;H|1\rangle =\frac{1}{\sqrt{2}}|0\rangle -\frac{1}{\sqrt{2}}|1\rangle =|-\rangle .$$

What makes quantum operations remarkable is that they can reverse this process perfectly: applying the Hadamard transformation twice returns the qubit to its original state

(7) H|+〉=|0〉,H|−〉=|1〉.

This is fundamentally different from classical randomization, where a process that generates a fair coin toss cannot be “undone” to recover the original definite state. This process, entering a fully indeterminate state and returning to a definite one, is not possible in classical systems. In the classical case, generating a random bit can be mathematically described as the action of a stochastic matrix on the state |0〉 or |1〉 (8). A stochastic matrix is defined as a matrix whose entries are all nonnegative real numbers and whose columns each sum to one. Specifically, a matrix whose entries are all 1/2 maps both |0〉 and |1〉 into a uniformly distributed random bit,

(8) $$\left(\begin{array}{cc} \frac{1}{2} & \frac{1}{2}\\ \frac{1}{2} & \frac{1}{2} \end{array}\right)|i\rangle =\left(\begin{array}{c} \frac{1}{2}\\ \frac{1}{2} \end{array}\right),\;\mathrm{for}\;i\in \left\{0,1\right\}.$$

Applying this matrix again will not make the random bit deterministic. It reflects the fundamental reversibility and coherence of quantum operations, which have no classical counterpart.

### 3.3. Multi-Qubit States and Independence

When dealing with more than one qubit, the joint state of the system is built by combining the individual qubit states through the tensor product. If a two-qubit state can be written as the direct product of two single-qubit states, the qubits are independent (9). In such cases, measuring one qubit does not provide any information about the other, just as in classical probability when two random variables are independent. Given two vectors $|\phi \rangle =\alpha_{0}|0\rangle +\alpha_{1}|1\rangle$ and $|\psi \rangle =\beta_{0}|0\rangle +\beta_{1}|1\rangle$, the tensor product |ϕ〉⊗|ψ〉 is defined as

(9) $$|\phi \rangle ⊗|\psi \rangle =(\alpha_{0}|0\rangle +\alpha_{1}\left|1\rangle \right)⊗(\beta_{0}|0\rangle +\beta_{1}\left|1\rangle \right)=\alpha_{0}\beta_{0}|00\rangle +\alpha_{0}\beta_{1}|01\rangle +\alpha_{1}\beta_{0}|10\rangle +\alpha_{1}\beta_{1}|11\rangle .$$

For example, suppose that each qubit has its own probability of producing 0 or 1, regardless of the other. The probability P(X=i) of obtaining the value i∈{0,1} on the first qubit is given by the sum of the absolute values squared of the components |i0〉 and |i1〉 of (9). Similarly, the probability P(Y=j) of measuring the second qubit is given by the sum of the absolute values squared of the components |0j〉 and |1j〉 of (9). Then, knowing that the measurement result of the second qubit has no effect on predicting the outcome of the first (10).

(10) $$P\left(X=0|Y=0\right)=\frac{P(X=0,Y=0)}{P(Y=0)}=\frac{|\alpha_{0}\beta_{0}{|}^{2}}{|\alpha_{0}\beta_{0}{|}^{2}+{\left|\alpha_{1}\beta_{0}\right|}^{2}}={\left|\alpha_{0}\right|}^{2}.$$

### 3.4. Entanglement and Strategic Correlation

Quantum mechanics also allows for entangled states, which cannot be expressed as products of independent qubits. In an entangled pair, the measurement outcomes are strongly correlated, even if the qubits are far apart. A simple example is a state where both qubits are always found in the same value: either both 0 or both 1, with certain probabilities for each case (11).

(11) $$\alpha |00\rangle +\beta |11\rangle ,\;\alpha ,\beta \in C,\;\alpha ,\beta \neq {0,\;|\alpha |}^{2}+{\left|\beta \right|}^{2}=1.$$

Measuring one qubit immediately determines the value of the other (12).

(12) $$P\left(X=0|Y=0\right)=\frac{P(X=0,Y=0)}{P(Y=0)}=\frac{{\left|\alpha \right|}^{2}}{{\left|\alpha \right|}^{2}}=1.$$

The state in the form of (11) is a key component of the quantum game protocol and a necessary source of non-classical correlation between the players’ strategies.

In quantum game theory, and potentially in negotiation protocols, entanglement is a powerful resource. It creates correlations between participants’ strategies that cannot be explained by classical probability. This means that players can coordinate their actions in ways that classical systems cannot replicate, potentially altering equilibrium outcomes and enabling new forms of cooperative or competitive behavior.

### 3.5. The Eisert-Wilkens-Lewenstein Quantum Game Scheme

One of the most notable approaches to quantizing classical games was proposed by Eisert, Wilkens, and Lewenstein in [14]. Their approach transforms 2×2 bimatrix games by formulating them within a quantum-mechanical framework that incorporates entanglement and unitary operators as strategic choices. The Eisert–Wilkens–Lewenstein (EWL) framework aims to integrate the traditional game within the structure of quantum mechanics, ensuring that the classical version emerges as a specific instance while enabling the discovery of new equilibria, potentially leading to more cooperative outcomes than achievable through classical play.

#### 3.5.1. From Classical to Quantum Games

The scheme begins with a standard 2×2 bimatrix game (13), where each player chooses one of two actions or mixes between them probabilistically. The corresponding expected payoff functions (14) describe how a player’s payoff depends on the probability of each combination of actions being played. In essence, the formula multiplies each payoff entry by the probability of reaching it and sums the results. Let us consider a 2×2 bimatrix game in which player 1 and player 2 use mixed strategies (p,1−p) and (q,1−q), respectively

(13) $$\begin{array}{ccc} \; & q & 1-q\\ p & \left(a_{00},b_{00}\right) & \left(a_{01},b_{01}\right)\\ 1-p & \left(a_{10},b_{10}\right) & \left(a_{11},b_{11}\right) \end{array}.$$

The payoff functions of this game are given by

(14) $$u_{1(2)}\left(p,q\right)=a_{00}\left(b_{00}\right)pq+a_{01}\left(b_{01}\right)p\left(1-q\right)+a_{10}\left(b_{10}\right)\left(1-p\right)q+a_{11}\left(b_{11}\right)\left(1-p\right)\left(1-q\right).$$

#### 3.5.2. Step 1: Entanglement

The process begins by preparing a pair of qubits, one for each player, in a shared entangled state (16). This is done by applying the entangling operator *J* defined in (15), which links the qubits so that the players’ choices can later be correlated beyond what is possible classically.

(15) $$J=\left(1⊗1+i\sigma_{x}⊗\sigma_{x}\right)/\sqrt{2}=\frac{1}{\sqrt{2}}\left(\begin{array}{cccc} 1 & 0 & 0 & i\\ 0 & 1 & i & 0\\ 0 & i & 1 & 0\\ i & 0 & 0 & 1 \end{array}\right).$$

Applying *J* to |00〉 results in the state

(16) $$J|00\rangle =\frac{1}{\sqrt{2}}\left(|00\rangle +i|11\rangle \right).$$

The result is a state in which outcomes such as |00〉 and |11〉 occur in a superposition, with complex amplitudes rather than simple probabilities. Conceptually, this means that the game starts with built-in quantum correlation.

#### 3.5.3. Step 2: Quantum Strategies

Once the entangled state is prepared, Each player applies a unitary operator $U_{A},U_{B}\in \mathrm{SU}\left(2\right)$ to their respective qubit

(17) $$|\psi \rangle =\left(U_{A}⊗U_{B}\right)J|00\rangle .$$

These operations can be thought of as rotations in a two–dimensional complex space. A general form of such a unitary move is parameterized by three real numbers (18), which means that instead of being limited to two discrete strategies (or mixtures), players now have access to an infinite variety of possible moves.

(18) $$U\left(\theta ,\alpha ,\beta \right)=\left(\begin{array}{cc} e^{i\alpha }\cos\frac{\theta }{2} & ie^{i\beta }\sin\frac{\theta }{2}\\ ie^{-i\beta }\sin\frac{\theta }{2} & e^{-i\alpha }\cos\frac{\theta }{2} \end{array}\right),\;\theta \in \left[0,\pi \right],\alpha ,\beta \in \left[0,2\pi \right).$$

Another way to view these operations is to express them in terms of the identity 1 and the Pauli matrices *X*, *Y*, and *Z* as a basis

(19) $$U\left(\theta ,\alpha ,\beta \right)=1\cos\frac{\theta }{2}\cos\alpha +iX\sin\frac{\theta }{2}\cos\beta -iY\sin\frac{\theta }{2}\sin\beta +iZ\cos\frac{\theta }{2}\sin\alpha .$$

This emphasizes that a quantum strategy is not just a simple action but a transformation of a quantum state.

#### 3.5.4. Step 3: Measurement and Payoffs

After both players act, the entanglement is undone by applying the inverse entangling operator $J^{†}$ to (17)

(20) $$|\psi_{f}\rangle =J^{†}\left(U_{A}⊗U_{B}\right)J|00\rangle .$$

The payoff functions are determined by the amplitudes of $|\psi_{f}\rangle$ and the payoff matrix of the corresponding classical 2×2 game. If the state (20) is expressed in the form

(21) $$|\psi_{f}\rangle =\langle 00|\psi_{f}\rangle |00\rangle +\langle 01|\psi_{f}\rangle |01\rangle +\langle 10|\psi_{f}\rangle |10\rangle +\langle 11|\psi_{f}\rangle |11\rangle ,$$

then

(22) $$u_{1}\left(U_{1},U_{2}\right)=\sum_{i,j\in \{0,1\}}a_{ij}|\langle ij|\psi_{f}\rangle {|}^{2},\;u_{2}\left(U_{1},U_{2}\right)=\sum_{i,j\in \{0,1\}}b_{ij}{\left|\langle ij|\psi_{f}\rangle \right|}^{2},$$

where

(23) $$\begin{array}{cc} \; & |\langle 00|\psi_{f}\rangle {|}^{2}={\left(\cos\left(\alpha_{1}+\alpha_{2}\right)\cos\frac{\theta_{1}}{2}\cos\frac{\theta_{2}}{2}+\sin\left(\beta_{1}+\beta_{2}\right)\sin\frac{\theta_{1}}{2}\sin\frac{\theta_{2}}{2}\right)}^{2},\\ \; & |\langle 01|\psi_{f}\rangle {|}^{2}={\left(\cos\left(\alpha_{1}-\beta_{2}\right)\cos\frac{\theta_{1}}{2}\sin\frac{\theta_{2}}{2}+\sin\left(\alpha_{2}-\beta_{1}\right)\sin\frac{\theta_{1}}{2}\cos\frac{\theta_{2}}{2}\right)}^{2},\\ \; & |\langle 10|\psi_{f}\rangle {|}^{2}={\left(\sin\left(\alpha_{1}-\beta_{2}\right)\cos\frac{\theta_{1}}{2}\sin\frac{\theta_{2}}{2}+\cos\left(\alpha_{2}-\beta_{1}\right)\sin\frac{\theta_{1}}{2}\cos\frac{\theta_{2}}{2}\right)}^{2},\\ \; & |\langle 11|\psi_{f}\rangle {|}^{2}={\left(\sin\left(\alpha_{1}+\alpha_{2}\right)\cos\frac{\theta_{1}}{2}\cos\frac{\theta_{2}}{2}-\cos\left(\beta_{1}+\beta_{2}\right)\sin\frac{\theta_{1}}{2}\sin\frac{\theta_{2}}{2}\right)}^{2}. \end{array}$$

The expected payoffs are then calculated as in (22) and (23), where each original payoff entry is weighted by the squared magnitude of the corresponding amplitude in the final quantum state. Put simply, instead of multiplying payoffs by classical probabilities, they are multiplied by probabilities generated through quantum measurement. This abundance of strategies is disadvantageous for game applications for two reasons. First, it is difficult to interpret these three strategy parameters. Second, and more importantly, quantum games, except in trivial cases, do not have Nash equilibria for such defined strategies. Nash equilibria are natural solutions in the practical applications of game theory.

Thus, a straightforward method is to establish a quantized game by providing players with a limited set of strategies, $\left\{U_{i}:i=1,\ldots ,n\right\}$, for n=2,3,4, each having specific $\left(\theta_{i},\alpha_{i},\beta_{i}\right)$ parameters (18). The quantized game bimatrix can then be represented as follows:

(24) $$\begin{array}{ccccc} \; & U_{1} & U_{2} & \ldots & U_{n}\\ U_{1} & \left(u_{1},u_{2}\right)\left(U_{1},U_{1}\right) & \left(u_{1},u_{2}\right)\left(U_{1},U_{2}\right) & \ldots & \left(u_{1},u_{2}\right)\left(U_{1},U_{n}\right)\\ U_{2} & \left(u_{1},u_{2}\right)\left(U_{2},U_{1}\right) & \left(u_{1},u_{2}\right)\left(U_{2},U_{2}\right) & \ldots & \left(u_{1},u_{2}\right)\left(U_{2},U_{n}\right)\\ \vdots & \vdots & \vdots & \ddots & \vdots \\ U_{n} & \left(u_{1},u_{2}\right)\left(U_{n},U_{1}\right) & \left(u_{1},u_{2}\right)\left(U_{n},U_{2}\right) & \ldots & \left(u_{1},u_{2}\right)\left(U_{n},U_{n}\right) \end{array}$$

As in the classic game, players can use these strategies as pure strategies or probabilistically mixed with coefficients belonging to vectors

(25) $$p=\left(p_{1},p_{2},\ldots ,p_{n}\right)\;\mathrm{and}\;q=\left(q_{1},q_{2},\ldots ,q_{n}\right),$$

where $p_{i},q_{i}\geq 0$ and $\sum_{i=1}^{n}p_{i}=\sum_{i=1}^{n}q_{i}=1$. Let’s assume that the players can now make decisions with a quantum device (black box) that implements the EWL quantization (24) of the game (13). By using strategies $U_{A},U_{B}\in \left\{U_{1},U_{2},\ldots ,U_{n}\right\}$ players operate in the space of qubit pairs {|i,j〉:i,j=0,1}, whose pair numbering |0,0〉, |0,1〉, |1,0〉 and |1,1〉 corresponds to the position of the elements of the classical payoff matrix (13). The qubits |i,j〉 determine the player’s decision just as in the classical game, regardless of how many $U_{i}$ strategies each player has available.

### 3.6. Embedding the Classical Game

A crucial property of the EWL scheme is that the classical game is fully embedded within it. By restricting the allowed quantum strategies to two particular unitary operations, the identity and a Pauli transformation, the expected payoffs (27) reduce exactly to the classical mixed–strategy payoffs (14). In this way, the EWL framework extends the classical game without replacing it.

Consider the form of the game (24) for n = 2, with operators from (18) restricted to α,β=0. Thus, formulas (23) reduce to

(26) $$\begin{array}{ccc} \; & |\langle 00|\psi_{f}\rangle {|}^{2}=\cos^{2}\frac{\theta_{1}}{2}\cos^{2}\frac{\theta_{2}}{2}, & |\langle 01|\psi_{f}\rangle {|}^{2}=\cos^{2}\frac{\theta_{1}}{2}\sin^{2}\frac{\theta_{2}}{2},\\ \; & |\langle 10|\psi_{f}\rangle {|}^{2}=\sin^{2}\frac{\theta_{1}}{2}\cos^{2}\frac{\theta_{2}}{2}, & |\langle 11|\psi_{f}\rangle {|}^{2}=\sin^{2}\frac{\theta_{1}}{2}\sin^{2}\frac{\theta_{2}}{2}. \end{array}$$

The payoff functions (22) then take the form

(27) $$\begin{array}{c} u_{1(2)}\left(U_{1},U_{2}\right)=a_{00}\left(b_{00}\right)\cos^{2}\frac{\theta_{1}}{2}\cos^{2}\frac{\theta_{2}}{2}+a_{01}\left(b_{01}\right)\cos^{2}\frac{\theta_{1}}{2}\sin^{2}\frac{\theta_{2}}{2}\\ \;\;\;\;\;\;\;\;\;\;\;\;\;+a_{10}\left(b_{10}\right)\sin^{2}\frac{\theta_{1}}{2}\cos^{2}\frac{\theta_{2}}{2}+a_{11}\left(b_{11}\right)\sin^{2}\frac{\theta_{1}}{2}\sin^{2}\frac{\theta_{2}}{2}. \end{array}$$

By substituting $\cos^{2}\frac{\theta_{1}}{2}=p$ and $\cos^{2}\frac{\theta_{2}}{2}=q$ into Equation (27), we retrieve the classical expected payoff (14) linked to mixed-strategy play in game (13). Player’s quantum strategies, which replicate their classical actions, are linked to the unitary operators: $U_{1}=U\left(0,0,0\right)=1$ and $U_{2}=U\left(\pi ,0,0\right)=iX$. When players implement these strategies, the game’s quantization (24) reduces to

(28) $$\begin{array}{ccc} \; & 1 & iX\\ 1 & \left(a_{00},b_{00}\right) & \left(a_{01},b_{01}\right)\\ iX & \left(a_{10},b_{10}\right) & \left(a_{11},b_{11}\right) \end{array},$$

i.e., is identical to the classical game from (13). Quantum players have the option to consider *I* and iX as pure strategies or to employ mixed strategies by selecting them with certain probabilities. Moreover, they may not realize that the game’s mechanism is quantum and might employ quantum strategies similarly to classical ones, utilizing the quantum computer as a black box for choosing strategies $U_{1},U_{2},\ldots ,U_{n}$ as if they were classical.

To make analysis feasible, one can also restrict the continuous family of quantum strategies to a finite set (24). This produces a quantized version of the bimatrix game, with cells corresponding to pairs of quantum strategies. Players may use these strategies purely or in probabilistic mixtures, as in the classical case (25). Moreover, they may not realize the game’s mechanism is quantum and might employ quantum strategies in the same way as classical ones, effectively using a quantum computer as a black box for choosing strategies $U_{1},U_{2},\ldots ,U_{n}$ as if they were classical.

### 3.7. Conceptual Significance

From the perspective of game theory, the EWL scheme is important because it expands the strategy space while preserving the logical structure of the original game. The players’ payoffs are still determined by the same matrix entries, but the probabilities that decide which entry is realized are now generated through quantum mechanics.

This enrichment of the strategy space can lead to new equilibria not present in the classical model. In particular, dilemmas that seem unavoidable in the classical setting may be resolved in the quantum realm.

A particularly important class of such quantum extensions is those that are *invariant with respect to isomorphic transformations of the original game*. These extensions ensure that the quantized game preserves the symmetry structure of the classical one, so that the quantum version truly represents the same strategic problem. Recent studies have analyzed these permissible extensions in greater detail, both in the case of three-strategy quantum games [38] and in four-strategy quantum extensions [39].

In summary, the Eisert–Wilkens–Lewenstein protocol provides a bridge between classical and quantum games. It preserves the structure of classical payoffs (13) and (14), introduces new strategic degrees of freedom through unitary operations (18), (24), and recovers the classical game when restricted to specific moves (28). For readers familiar with game theory but not quantum mechanics, one can think of it as taking the familiar logic of a bimatrix game and embedding it into a larger framework, where correlations and strategies are enhanced by the rules of quantum mechanics.

In the next sections, we will explore the quantization of classical games described by (24), where the initial two strategies mirror classical ones $U_{1}=1$ and $U_{2}=iX$, while the additional strategies correspond to selected unitary operators.

## 4. Cooperation vs. Competition

One of the most prevalent ethical dilemmas in many negotiation games is the tension between cooperation and competition. While cooperation allows negotiators to maximize joint gains and create value through integrative solutions, the competitive drive to secure individual advantage can undermine these collective benefits. Game-theoretic models, such as the Prisoner’s Dilemma and various bargaining games, illustrate how rational actors, motivated by self-interest, may forgo mutually beneficial outcomes in favor of strategies that protect or advance their own positions, often at the expense of the group as a whole. This dilemma raises important ethical questions about the responsibilities negotiators have, not only to themselves but also to their counterparts and the wider community impacted by the results of their negotiations.

### Prisoner’s Dilemma

The Prisoner’s Dilemma is a foundational concept in classical game theory, first formulated in the 1950s by Merrill Flood and Melvin Dresher [40] and later popularized by Albert W. Tucker [41]. It models a situation where two rational agents, acting in their own self-interest, end up in a suboptimal outcome despite the possibility of mutual cooperation. The standard setup involves two players who must independently choose whether to cooperate or defect, with the payoff structure designed so that unilateral defection yields a higher individual reward, while mutual cooperation is collectively better than mutual defection.

The game can be depicted by a 2×2 bimatrix in Table 1.

In this game, the unique Nash equilibrium is mutual defection because both players possess a dominant strategy to defect, irrespective of the other’s decision. However, this leads to a reduced payoff for both players (1,1) compared to mutual cooperation (3,3), highlighting a core conflict between individual rational behavior and collective benefit. This conundrum has been extensively explored in the realms of ethics, trust, and social cooperation, challenging the constraints of purely rational actions [42,43].

A promising approach to overcoming the limitations of the classical Prisoner’s Dilemma has been proposed within the framework of quantum game theory. Specifically, the quantum extension introduced by Eisert, Wilkens, and Lewenstein (EWL) in their seminal work [14] explores how the strategic space of players can be expanded by allowing quantum strategies implemented via unitary operations on entangled qubits representing player choices.

In the EWL model, players are no longer restricted to classical pure strategies (Cooperate or Defect) or even probabilistic combinations thereof but can choose unitary transformations that operate on their respective qubits. As shown in the previous section, the game starts with an entangled initial state (16), and after each player applies their chosen unitary strategy (18), a final measurement determines the outcome and corresponding payoffs (22). When players are restricted to a specific subset of unitary operations, the EWL framework enables the emergence of a new Nash equilibrium, with each player receiving a payoff equal to the mutual cooperation payoff of 3, an outcome that is Pareto superior to mutual defection with the mutual payoff of 1 [14]. This result demonstrates that, under quantum rules, rational agents may achieve higher collective and individual outcomes than those possible within the constraints of classical game theory.

Nevertheless, the EWL formulation has faced important critiques. Benjamin and Hayden [44] argued that the cooperative equilibrium observed in the EWL framework is a consequence of restricting the players’ strategies to a particular subset of unitary operations. Once the full set of local unitary strategies is allowed, the quantum game admits no new equilibria beyond those of the classical game, and mutual cooperation no longer emerges as a rational outcome. A complementary critique was presented by van Enk and Pike [45], who contend that the quantum version of the Prisoner’s Dilemma alters the structure of the original game by introducing fundamentally new strategies and payoff mappings. In their view, the “solution” provided by the EWL scheme does not resolve the dilemma within the classical game’s logic, but rather replaces it with a different game altogether, thus calling into question whether the dilemma has genuinely been overcome or merely reframed.

In the approach proposed in [38,39], both of the aforementioned criticisms are effectively addressed. To overcome the objection that the choice of players’ strategies in quantum games depends on an arbitrary selection of admissible unitary operations, we define the players’ strategy sets to be identical to those of mixed strategies in classical game theory. At the same time, the quantum nature of the game is preserved by embedding the game in the quantum domain and enriching the set of strategies with one or two additional, purely quantum operations that significantly expand the space of admissible outcomes while still imitating classical actions such as cooperation and defection. Furthermore, to ensure that the quantum game genuinely constitutes an extension of the classical game, rather than a different game altogether, we impose an invariance condition under isomorphic transformations of the classical game. This guaranties that the additional quantum operations are not arbitrary and provide structural continuity between the classical and quantum formulations, maintaining interpretability and consistency across both domains.

An instructive example of a quantum extension of the Prisoner’s Dilemma is obtained by augmenting the classical strategy set with a single additional unitary operation, as defined in Equation (32) of [38]. In this extension, players may choose either the classical strategies: cooperation and defection, represented respectively by 1 and iX operators, or a third, purely quantum strategy that is inaccessible in the classical framework. When both players adopt this quantum strategy, selected in an arbitrary way, the game, similarly to the EWL paper [14], reaches a Nash equilibrium that is also Pareto-optimal, with each player receiving a payoff equal to the mutual cooperation payoff of 3. However, this quantum extension breaks invariance under isomorphic transformations of the classical game. The introduction of the new strategy modifies the symmetry structure of the payoff matrix, effectively privileging a specific representation of the game. As a result, while it achieves cooperative outcomes, it no longer preserves the formal equivalence between classically isomorphic games.

In [38], it is further demonstrated that there exist quantum extensions of the original game that preserve invariance under isomorphic transformations of the classical Prisoner’s Dilemma while simultaneously admitting Nash equilibria with superior Pareto efficiency. An explicit example is given in Equation (29), where the strategy set, apart from classical actions 1 and iX, includes a balanced superposition of the identity and three Pauli matrices $U_{3}=\frac{1}{2}\left(1+iX+iY+iZ\right)$ applied by both players.

(29) $$\begin{array}{cccc} \; & 1 & iX & U_{3}\\ 1 & \left(3,3\right) & \left(0,5\right) & \left(\frac{9}{4},\frac{9}{4}\right)\\ iX & \left(5,0\right) & \left(1,1\right) & \left(\frac{9}{4},\frac{9}{4}\right)\\ U_{3} & \left(\frac{9}{4},\frac{9}{4}\right) & \left(\frac{9}{4},\frac{9}{4}\right) & \left(\frac{9}{4},\frac{9}{4}\right) \end{array}.$$

In this construction, there are three Nash equilibria in pure strategies $\left(iX,U_{3}\right)$, $\left(U_{3},iX\right)$ and $\left(U_{3},U_{3}\right)$ yielding a payoff of $\frac{9}{4}$ for both players, which is strictly greater than the punishment payoff 1. This outcome maintains the structural integrity of the classical game through isomorphism invariance and achieves a fair and symmetric cooperative solution that lies strictly above the classical equilibrium, thereby offering a compelling resolution of the dilemma within a fully consistent quantum framework.

## 5. Self-Interests vs. Equity

Negotiation games also frequently expose the ethical conflict between self-interest and equity. While game-theoretic reasoning encourages players to maximize their own payoffs, this pursuit can lead to highly unequal and sometimes exploitative outcomes, particularly when one party holds more power or information. Such outcomes may be efficient in the Pareto sense, but they often violate widely held norms of fairness and distributive justice. The resulting tension highlights a fundamental ethical dilemma: whether negotiators should accept less personally advantageous terms to ensure a more equitable allocation of resources, or prioritize their own gains even when it perpetuates or exacerbates inequality.

### 5.1. Ultimatum Game

The Ultimatum Game is a central experimental framework in behavioral economics and negotiation research, first developed in the early 1980s by Werner Güth, Rolf Schmittberger, and Bernd Schwarze [23]. It models a bargaining situation in which one player, the proposer, is allocated a fixed sum of resources and must decide how to divide it with a second player, the responder. The responder can either accept the offer, in which case the proposed division is implemented, or reject it, in which case both players receive nothing. Both the unfair division and the fair division are outcomes realized by Nash equilibria. Although the strategy pair consisting of player 1 making a fair offer and player 2 accepting the fair division while rejecting the unfair one constitutes a Nash equilibrium that yields equal payoffs, only the strategy pair in which player 1 makes an unfair offer and player 2 accepts every offer constitutes a Nash equilibrium that is subgame perfect. It is this latter outcome that is commonly regarded as the rational solution from the perspective of game theory. However, empirical studies consistently find that low offers are frequently rejected, indicating that considerations of fairness, reciprocity, and social norms influence decision-making. From an ethical perspective, the game’s equilibrium outcome, accepting and offering the smallest positive amount, invites reflection on distributive justice, the morality of exploiting positional advantage, and the extent to which strategic self-interest can be reconciled with equitable treatment.

**Figure 1:**
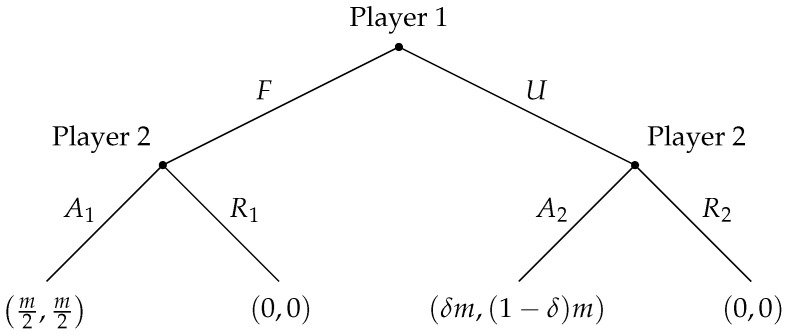
Ultimatum game.

Figure 1 presents the standard representation of the Ultimatum Game in extensive form. Player 1 (the proposer) chooses between a fair split *F*, allocating $\frac{m}{2}$ to each player, and an unequal split *U*, offering the responder only a share (1−δ)m with 0.5<δ<1. Player 2 (the responder) then decides whether to accept ($A_{1}$ or $A_{2}$), implementing the proposed division, or reject ($R_{1}$ or $R_{2}$), in which case both players receive nothing. This structure captures the essential strategic and ethical tension of the game: the trade-off between maximizing individual payoffs and proposing or accepting an allocation perceived as fair.

Normal form of the Ultimatum Game, showing the strategic interactions between the proposer (player 1) and the responder (player 2) in the classical setting, with payoffs corresponding to the outcomes of each possible combination of offers and responses.

(30) $$\begin{array}{ccccc} \; & A_{1},A_{2} & A_{1},R_{2} & R_{1},A_{2} & R_{1},R_{2}\\ F & \left(\frac{m}{2},\frac{m}{2}\right) & \left(\frac{m}{2},\frac{m}{2}\right) & \left(0,0\right) & \left(0,0\right)\\ U & \left(\delta m,(1-\delta )m\right) & \left(0,0\right) & \left(\delta m,(1-\delta )m\right) & \left(0,0\right) \end{array}$$

If player 1 uses a behavioral strategy (p,1−p) and player 2 uses $\left(\left(q_{1},1-q_{1}\right),\left(q_{2},1-q_{2}\right)\right)$ then the expected payoff resulting from playing these strategies is

(31) $$\begin{array}{cc} \; & u_{1}\left(\left(p,1-p\right),\left(\left(q_{1},1-q_{1}\right),\left(q_{2},1-q_{2}\right)\right)\right)=\frac{m}{2}pq_{1}+\delta m\left(1-p\right)q_{2},\\ \; & u_{2}\left(\left(p,1-p\right),\left(\left(q_{1},1-q_{1}\right),\left(q_{2},1-q_{2}\right)\right)\right)=\frac{m}{2}pq_{1}+\left(1-\delta \right)m\left(1-p\right)q_{2}. \end{array}$$

Although the Ultimatum Game differs significantly in structure from a 2×2 bimatrix game, we can still make use of the general idea behind the EWL scheme and adapt the model to incorporate unitary strategies.

Let us first observe that player 1 has one information set, while player 2 has two information sets in the game shown in Figure 1. This observation corresponds to modeling player 1 as acting on one qubit and player 2 as acting on two qubits.

The players begin with a specially prepared three qubit entangled state that maximizes the correlations among all three qubits. Each player then performs their move: player 1 applies a unitary operation to their qubit, while player 2 applies a unitary operation to each of their two qubits. After all moves are made, the entangled state is disentangled using the inverse of the initial entangling operation, preparing it for measurement. The final state of the system determines the probabilities of different measurement outcomes, which, in turn, define the players’ payoffs according to the structure of the game. These payoffs directly depend on the quantum operations chosen by the players and on how those operations have affected the joint state of the system. A shared entangled state in this case is

(32) $$|\psi_{0}\rangle =J^{\prime }|000\rangle ,$$

where $J^{\prime }$ is defined as

(33) $$J^{\prime }=\left(1⊗1⊗1+i\sigma_{x}⊗\sigma_{x}⊗\sigma_{x}\right)/\sqrt{2}.$$

The action of the operator $J^{\prime }$ on |000〉 results in

(34) $$J|000\rangle =\frac{1}{\sqrt{2}}(|000\rangle +i|111\rangle ).$$

Player 1 acts with a unitary operator $U_{A}$ on the first qubit, and Player 2 applies $U_{B_{1}}$ to the second qubit and $U_{B_{2}}$ to the third,

(35) $$|\psi \rangle =U_{A}⊗U_{B_{1}}⊗U_{B_{2}}|\psi_{0}\rangle .$$

Analogously to (20), we construct the final state $|\psi_{f}\rangle =J^{\prime †}\left(U_{A}⊗U_{B_{1}}⊗U_{B_{2}}\right)J^{\prime }|000\rangle$, which will be used to define the payoff functions. Let

(36) $$\begin{array}{cc} \; & u_{1}\left(U_{A},U_{B_{1}},U_{B_{2}}\right)=\frac{m}{2}\left(|\langle 000|\psi_{f}\rangle {|}^{2}+{\left|\langle 001|\psi_{f}\rangle \right|}^{2}\right)+\delta m\left(|\langle 100|\psi_{f}\rangle {|}^{2}+{\left|\langle 110|\psi_{f}\rangle \right|}^{2}\right),\\ \; & u_{2}\left(U_{A},U_{B_{1}},U_{B_{2}}\right)=\frac{m}{2}\left(|\langle 000|\psi_{f}\rangle {|}^{2}+{\left|\langle 001|\psi_{f}\rangle \right|}^{2}\right)+\left(1-\delta \right)m\left(|\langle 100|\psi_{f}\rangle {|}^{2}+{\left|\langle 110|\psi_{f}\rangle \right|}^{2}\right). \end{array}$$

One can verify that

(37) $$\begin{array}{cc} \; & |\langle 000|\psi_{f}\rangle {|}^{2}=\cos^{2}\left(\alpha_{1}+\alpha_{2}+\alpha_{3}\right)\cos^{2}\frac{\theta_{1}}{2}\cos^{2}\frac{\theta_{2}}{2}\cos^{2}\frac{\theta_{3}}{2}+\sin^{2}\left(\beta_{1}+\beta_{2}+\beta_{3}\right)\sin^{2}\frac{\theta_{1}}{2}\sin^{2}\frac{\theta_{2}}{2}\sin^{2}\frac{\theta_{3}}{2},\\ \; & |\langle 001|\psi_{f}\rangle {|}^{2}=\cos^{2}\left(\alpha_{1}+\alpha_{2}-\beta_{3}\right)\cos^{2}\frac{\theta_{1}}{2}\cos^{2}\frac{\theta_{2}}{2}\sin^{2}\frac{\theta_{3}}{2}+\sin^{2}\left(\beta_{1}+\beta_{2}-\alpha_{3}\right)\sin^{2}\frac{\theta_{1}}{2}\sin^{2}\frac{\theta_{2}}{2}\cos^{2}\frac{\theta_{3}}{2},\\ \; & |\langle 100|\psi_{f}\rangle {|}^{2}=\cos^{2}\left(-\beta_{1}+\alpha_{2}+\alpha_{3}\right)\sin^{2}\frac{\theta_{1}}{2}\cos^{2}\frac{\theta_{2}}{2}\cos^{2}\frac{\theta_{3}}{2}+\sin^{2}\left(\alpha_{1}-\beta_{2}-\beta_{3}\right)\cos^{2}\frac{\theta_{1}}{2}\sin^{2}\frac{\theta_{2}}{2}\sin^{2}\frac{\theta_{3}}{2},\\ \; & |\langle 110|\psi_{f}\rangle {|}^{2}=\cos^{2}\left(-\beta_{1}-\beta_{2}+\alpha_{3}\right)\sin^{2}\frac{\theta_{1}}{2}\sin^{2}\frac{\theta_{2}}{2}\cos^{2}\frac{\theta_{3}}{2}+\sin^{2}\left(\alpha_{1}+\alpha_{2}-\beta_{3}\right)\cos^{2}\frac{\theta_{1}}{2}\cos^{2}\frac{\theta_{2}}{2}\sin^{2}\frac{\theta_{3}}{2}. \end{array}$$

Similarly to the quantum model for a 2×2 game, the payoff function (36) reduces to the classical payoff under the constraints $\alpha_{i}=\beta_{i}=0$ for i∈{1,2,3}. Indeed,

(38) $$\begin{array}{cc} \; & u_{1}\left(U_{A}\left(\theta_{1},0,0\right),U_{B_{1}}\left(\theta_{2},0,0\right),U_{B_{2}}\left(\theta_{3},0,0\right)\right)=\frac{m}{2}\cos^{2}\frac{\theta_{1}}{2}\cos^{2}\frac{\theta_{2}}{2}+\delta m\sin^{2}\frac{\theta_{1}}{2}\cos^{2}\frac{\theta_{3}}{2},\\ \; & u_{2}\left(U_{A}\left(\theta_{1},0,0\right),U_{B_{1}}\left(\theta_{2},0,0\right),U_{B_{2}}\left(\theta_{3},0,0\right)\right)=\frac{m}{2}\cos^{2}\frac{\theta_{1}}{2}\cos^{2}\frac{\theta_{2}}{2}+\left(1-\delta \right)m\sin^{2}\frac{\theta_{1}}{2}\cos^{2}\frac{\theta_{3}}{2}. \end{array}$$

It is easy to see that formula (38) coincides with (31) when we make the substitution $\cos^{2}\frac{\theta_{1}}{2}=p$, $\cos^{2}\frac{\theta_{2}}{2}=q_{1}$, $\cos^{2}\frac{\theta_{3}}{2}=q_{2}$.

The quantum model allows us to examine various forms of quantum extensions. Of particular interest is the extension of the classical game by incorporating one unitary strategy. Let us first note that the unitary strategies 1 and iX can be identified with the classical actions in the Ultimatum game. Keeping in mind that 1=U(0,0,0) and iX=U(π,0,0), it follows from (38) that

(39) $$\begin{array}{cc} \; & u_{1}\left(1,1,1\right)=u_{1}\left(1,1,iX\right)=\frac{m}{2}\\ \; & u_{1}\left(1,iX,1\right)=u_{1}\left(1,iX,iX\right)=u_{1}\left(iX,1,1\right)=u_{1}\left(iX,iX,iX\right)=0,\\ \; & u_{1}\left(iX,1,1\right)=u_{1}\left(iX,iX,1\right)=\delta m. \end{array}$$

Analogously, we determine the payoff values for the second player. Hence, the matrix form based on (39) is

(40) $$\begin{array}{ccccc} \; & 1,1 & 1,iX & iX,1 & iX,iX\\ 1 & \left(\frac{m}{2},\frac{m}{2}\right) & \left(\frac{m}{2},\frac{m}{2}\right) & \left(0,0\right) & \left(0,0\right)\\ iX & \left(\delta m,(1-\delta )m\right) & \left(0,0\right) & \left(\delta m,(1-\delta )m\right) & \left(0,0\right) \end{array},$$

which coincides with (30).

Let us now consider the case in which the players have an additional unitary strategy U(0,π/2,0)=iZ at their disposal. Then

(41) $$\begin{array}{cc} u_{1}\left(1,1,iZ\right) & =u_{1}\left(1,iX,iZ\right)=u_{1}\left(1,iZ,1\right)=u_{1}\left(iX,1,iZ\right)=u_{1}\left(iX,iZ,1\right)=u_{1}\left(iX,iZ,iX\right)\\ \; & =u_{1}\left(iZ,1,1\right)=u_{1}\left(iZ,iX,1\right)=u_{1}\left(iZ,iX,iZ\right)=u_{1}\left(iZ,iZ,iZ\right)=0,\\ u_{1}\left(1,iZ,iZ\right) & =u_{1}\left(iX,iX,iZ\right)=u_{1}\left(iZ,1,iZ\right)=u_{1}\left(iZ,iZ,1\right)=u_{1}\left(iZ,iZ,iX\right)=\frac{m}{2},\\ u_{1}\left(1,iZ,iX\right) & =u_{1}\left(iX,iZ,iZ\right)=u_{1}\left(iZ,1,iX\right)=u_{1}\left(iZ,iX,iX\right)=\delta m. \end{array}$$

By determining in a similar manner the payoffs for player 2, we obtain the following game matrix:

(42) $$\begin{array}{cccccccccc} \; & 1,1 & 1,iX & 1,iZ & iX,1 & iX,iX & iX,iZ & iZ,1 & iZ,iX & iZ,iZ\\ 1 & \left(\frac{m}{2},\frac{m}{2}\right) & \left(\frac{m}{2},\frac{m}{2}\right) & \left(0,0\right) & \left(0,0\right) & \left(0,0\right) & \left(0,0\right) & \left(0,0\right) & \left(\delta m,(1-\delta )m\right) & \left(\frac{m}{2},\frac{m}{2}\right)\\ iX & \left(\delta m,(1-\delta )m\right) & \left(0,0\right) & \left(0,0\right) & \left(\delta m,(1-\delta )m\right) & \left(0,0\right) & \left(\frac{m}{2},\frac{m}{2}\right) & \left(0,0\right) & \left(0,0\right) & \left(\delta m,(1-\delta )m\right)\\ iZ & \left(0,0\right) & \left(\delta m,(1-\delta )m\right) & \left(\frac{m}{2},\frac{m}{2}\right) & \left(0,0\right) & \left(\delta m,(1-\delta )m\right) & \left(0,0\right) & \left(\frac{m}{2},\frac{m}{2}\right) & \left(\frac{m}{2},\frac{m}{2}\right) & \left(0,0\right) \end{array}$$

Extending the Ultimatum Game with the strategy iZ changes the course of the game and its final outcome with respect to the Nash equilibrium. Game (42) has three pure Nash equilibria (iX,iX,iZ), (iZ,1,iZ) and (iZ,iZ,1), each involving a quantum action and yielding a fair payoff of m/2 for each player.

The introduction of quantum strategies fundamentally reshapes the strategic landscape of the Ultimatum Game. In the classical version, the only subgame perfect equilibrium predicts the smallest possible offer being accepted, a solution that is strategically stable yet often regarded as ethically problematic due to its disregard for distributive fairness. By contrast, the quantum extension with access to the additional unitary strategy iZ generates multiple pure-strategy Nash equilibria: (iX,iX,iZ), (iZ,1,iZ), and (iZ,iZ,1) that yield equal payoffs of m/2 for both players. These outcomes preserve equilibrium stability while aligning more closely with fairness norms and principles of distributive justice. From an ethical standpoint, the quantum formulation therefore expands the set of viable solutions to include equilibria that are both strategically rational and socially desirable, illustrating how quantum game theory can reconcile self-interest with equitable treatment in bargaining scenarios.

### 5.2. Battle of the Sexes

The Battle of the Sexes is a classic two-player coordination game in game theory, traditionally used to illustrate the problem of strategic decision-making when players prefer to coordinate but disagree on the preferred outcome. The game was first introduced under this name in the 1950s, often attributed to R. Duncan Luce and Howard Raiffa’s foundational work Games and Decisions (1957) [46], where it appeared as an example of games with multiple Nash equilibria and asymmetric preferences. The metaphor involves two players, typically referred to as Alice and Bob, who wish to meet but differ in their ideal choice of activity (e.g., ballet vs. fight), resulting in a coordination dilemma.

The game admits two pure-strategy Nash equilibria: one in which both players choose Ballet, favoring Alice, and another in which both choose Fight, favoring Bob. The specific payoff structure of this game is presented in Table 2. A mixed-strategy equilibrium also exists where both players randomize: Alice opts for $\frac{3}{4}B+\frac{1}{4}F$, and Bob chooses $\frac{1}{4}B+\frac{3}{4}F$, resulting in equal payoffs of $\left(\frac{3}{2},\frac{3}{2}\right)$ for both. The core dilemma lies not in a conflict of interest per se, since both players prefer coordination over misalignment, but in the difficulty of achieving consensus when their preferences diverge. The Battle of the Sexes thus illustrates the problem of equilibrium selection in asymmetric coordination games, which is particularly relevant in contexts like social conventions, standardization, or decision-making under uncertainty. Such dilemmas motivate the exploration of enhanced coordination mechanisms, including quantum strategies, to resolve preference asymmetry or enable fairer outcomes.

To address these challenges, we suggest an extension of the 4×4 game into the quantum domain, aligned with the framework described in [39]. The class $A_{1}$ extension for the parameter $\alpha_{1}=\frac{\pi }{4}$ introduces new unitary strategies $U_{3}=\left(1+iZ\right)/\sqrt{2}$ and $U_{4}=i\left(X+Y\right)/\sqrt{2}$, defined by the Pauli matrices *Y* and *Z*. Consequently, the game expansion from Table 2 manifests as follows:

(43) $$\begin{array}{ccccc} \; & 1 & iX & U_{3} & U_{4}\\ 1 & \left(3,2\right) & \left(1,1\right) & \left(\frac{5}{2},\frac{5}{2}\right) & \left(\frac{1}{2},\frac{1}{2}\right)\\ iX & \left(0,0\right) & \left(2,3\right) & \left(\frac{1}{2},\frac{1}{2}\right) & \left(\frac{5}{2},\frac{5}{2}\right)\\ U_{3} & \left(\frac{5}{2},\frac{5}{2}\right) & \left(\frac{1}{2},\frac{1}{2}\right) & \left(2,3\right) & \left(0,0\right)\\ U_{4} & \left(\frac{1}{2},\frac{1}{2}\right) & \left(\frac{5}{2},\frac{5}{2}\right) & \left(1,1\right) & \left(3,2\right) \end{array}.$$

**Figure 2:**
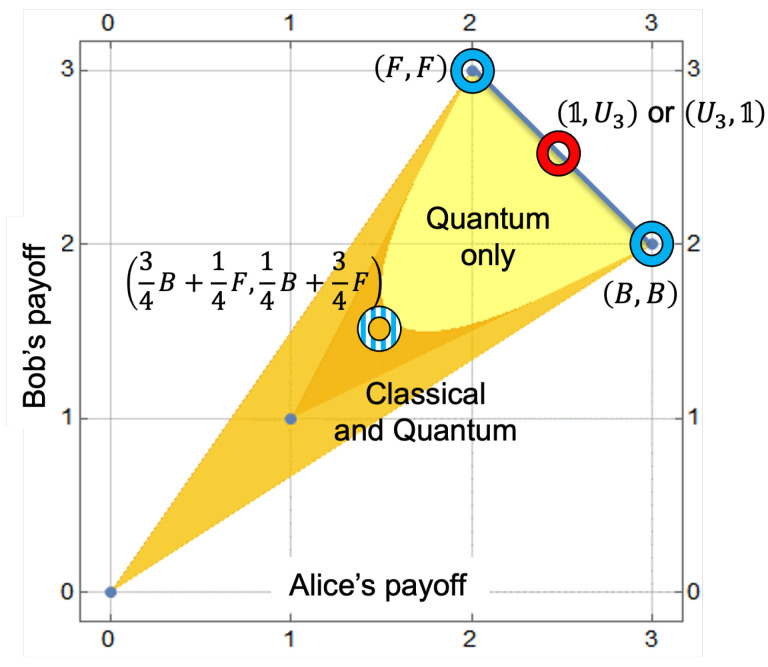
Feasible payoff regions in the Battle of the Sexes: Gold areas cover outcomes attainable in both classical and quantum versions, yellow highlights payoffs unique to the quantum game. Blue rings mark three classical NE (the equilibrium in mixed strategies is a striped pattern), while the red ring indicate NE of the quantum game. Blue line is the Pareto front.

The obtained extension matrix (43) provides a comprehensive solution to the problem presented in Table 2. Players are now presented with two Nash equilibria in pure strategies $\left(1,U_{3}\right)$ and $\left(U_{3},1\right)$, which are characterized by equal payoffs of $\frac{5}{2}$ for both players. This indicates that the initial disparity in payoff amounts will be addressed at the mean level of these two payoffs (2 and 3); see Figure 2.

This solution is Pareto optimal, thus solving the core issue of the Battle of the Sexes game. As in the Prisoner’s Dilemma, these solutions are a consequence of quantum randomization, the collapse of a mixed quantum state, which results in the selection of one of the strategy profiles (B,B) or (F,F). This dynamic can be likened to a mechanism in which Alice and Bob agree to flip a coin and, contingent on the outcome, select one of these strategy profiles. In this instance, however, the role of the coin is supplanted by a quantum mechanism. Note that the quantum version of the game (43) has a larger range of possible payoffs than the classical game. The yellow-marked area in Figure 2 includes payoffs that are impossible to obtain in the classical game [47].

The quantum extension resolves the initial disparity in payoffs, replacing classical randomization with quantum randomization, which enables outcome symmetry without sacrificing coordination. The graphical analysis shows that the quantum game’s feasible payoff set strictly contains the classical one: the gold areas represent outcomes available in both versions, and the yellow areas are unique to the quantum case. Classical and quantum equilibria are identified, with quantum solutions providing both fairness (equity) and Pareto efficiency.

Taken together, the quantum versions of the Battle of the Sexes and the Ultimatum Game illustrate how quantization can reshape distributive conflicts. In the classical formulations, stable outcomes either privilege one side’s preferences or permit inequitable offers to persist as equilibria. By contrast, quantum strategies expand the solution space to include equilibria that are more balanced, symmetrizing payoffs in coordination games and discouraging the acceptance of unfair offers in bargaining games. In this way, the quantum perspective shows how the persistent tension between self-interest and equity can be moderated, enabling fairer outcomes without sacrificing strategic stability.

## 6. Honesty vs. Deception

The tension between honesty and deception represents another central ethical dilemma in negotiation games. While transparency and truthful communication foster trust and facilitate fair agreements, game-theoretic analysis often shows incentives for strategic misrepresentation or withholding information to secure a better outcome. Such tactics may be rational and effective from a purely self-interested standpoint, but they can erode trust, damage relationships, and compromise the legitimacy of negotiated agreements. This dilemma emphasizes the challenge negotiators face in balancing the short-term gains often associated with deception against the ethical imperative and long-term benefits of honesty and integrity.

### Buyer–Seller Game

A classic setting for the honesty–deception dilemma is the buyer–seller game, where one side holds private information about value and the other must decide whether to trust it. This makes it a natural model for examining how quantum strategies might alter incentives for truth-telling.

The model describes two decision makers entering into price negotiations [48]. Suppose that the reservation prices of the buyer and seller can take one of two values

(44) $$r_{b}\in \left\{\frac{1}{2},\frac{3}{2}\right\}\;r_{s}\in \left\{0,1\right\},$$

respectively, with equal probability. The players know their reservation values but do not reveal them to their partners. Instead, they give declared values $r_{b}^{\prime }$ and $r_{s}^{\prime }$, taking the same possible values (44). Let us also assume that players use one of two strategies: a truth-telling strategy $\alpha_{i}$, i=b,s, which assumes that the declared values are equal to the actual values $r_{b}^{\prime }=r_{b}$, $r_{s}^{\prime }=r_{s}$, and the deception (lowball/highball) strategy $\beta_{i}$, which tries to understate the reported buying price $r_{b}^{\prime }=1/2$, regardless of actual $r_{b}$, or overstate the reported selling price $r_{s}^{\prime }=1$, regardless of actual $r_{s}$. The negotiation process unfolds in the following manner: both parties involved in the bargaining simultaneously declare their reservation prices $r_{i}^{\prime }$ (make offers at the same time). In the event that $r_{b}^{\prime }\geq r_{s}^{\prime }$, the transaction is considered completed, and the agreed price is determined as the average of the declared values $r=(r_{b}^{\prime }+r_{s}^{\prime })/2$. The buyer’s payoff is then equal to the excess between his limit of concessions and the negotiated price $r_{b}-r$. Conversely, the seller’s payoff is equivalent to the difference between the negotiated price and his limit of concessions, $r-r_{s}$. On the other hand, if $r_{b}^{\prime }<r_{s}^{\prime }$ the transaction is not concluded, then both payoffs are zero. The normal form of the game is exhibited in Table 3, where the units are 1/16 of a unit payoff of (44).

The game has two pure strategy Nash equilibria: $\left(\alpha_{b},\beta_{s}\right)$ and $\left(\beta_{b},\alpha_{s}\right)$. These equilibria are achieved when one player employs an unfair (lowball/highball) strategy and the other a truth-telling strategy; the former then receives a payoff of 6, and the latter a payoff of 2. Yet, if both parties adopt the unfair strategy $\left(\beta_{b},\beta_{s}\right)$, no transaction occurs, resulting in a payoff of 0 for each. They will get the best payoff (5,5) when they both use the strategy $\left(\alpha_{b},\alpha_{s}\right)$ of telling the truth, but this strategy pair is not an equilibrium. The game has one mixed strategy equilibrium that players can achieve by playing $\frac{2}{3}\alpha_{p}+\frac{1}{3}\beta_{p}$ and p=b,s; the payoffs for this strategy are equal to (4,4).

The space of possible strategies in the quantum game is much richer than the space of mixed strategies in the classical game. Consequently, quantum players possess a wider range of strategies, specifically unitary operators, that can be used. Suppose, for example, that players add a quantum strategy in the form of a Pauli matrix $U_{3}=iZ=U\left(0,\pi /2,\pi /2\right)$. When combined with the two “classical” strategies 1 and iX, it results in a novel game:

(45) $$\begin{array}{cccc} \; & 1 & iX & iZ\\ 1 & \left(5,5\right) & \left(2,6\right) & \left(0,0\right)\\ iX & \left(6,2\right) & \left(0,0\right) & \left(2,6\right)\\ iZ & \left(0,0\right) & \left(6,2\right) & \left(5,5\right) \end{array}.$$

The resulting game has a Nash equilibrium for the pair (iZ,iZ), and its payoff is the best possible in this game pair (5,5). Therefore, the incorporation of the extra unitary strategy iZ ensures that classical negotiators in the game outlined in Table 3 during the sales process lack motivation to overprice or underprice the deal, since the presence of strategy iZ deters both parties from choosing an alternative strategy, given that the other will also opt for iZ.

All classical games possess the characteristic that their solutions (Nash equilibria) do not alter regardless of the order in which players execute their strategies. For example, by switching both the rows and columns in Table 3, we can create an equivalent game where the equilibria continue to be the opposing strategies (highball/lowball) of the players. The game with exchanged strategies may also be extended by employing the unitary strategy iZ, similar to the approach in (45):

(46) $$\begin{array}{cccc} \; & 1 & iX & iZ\\ 1 & \left(0,0\right) & \left(6,2\right) & \left(5,5\right)\\ iX & \left(2,6\right) & \left(5,5\right) & \left(6,2\right)\\ iZ & \left(5,5\right) & \left(2,6\right) & \left(0,0\right) \end{array}.$$

However, this extended version does not resolve the initial problems efficiently, since its Nash equilibria (which occur only in mixed strategies) result in payoffs that are either uneven $\left(\frac{11}{2},\frac{7}{2}\right)$ or match those of the original game (4,4). In order to achieve equilibria that are as advantageous as those in game (45), it would be necessary to utilize, for instance, the iY operator in place of the iZ operator. Therefore, resolving the initial problem by including the unitary operator (5) does not provide a universal solution; the operator needs to be selected specifically to match how the game is structured, particularly the sequence of the players’ strategies. To address this issue, it is viable to utilize only those extensions that remain unchanged under isomorphic transformations of the original game. This concept has been analyzed in the studies [38,39].

An extension that remains invariant under any isomorphic transformations of the input game is the one involving two unitary strategies, as proposed in the work [39]. The extension matrix $A_{1}$, with the parameter $\alpha_{1}=\frac{\pi }{4}$, is specified by

(47) $$\begin{array}{ccccc} \; & 1 & iX & U_{3} & U_{4}\\ 1 & \left(5,5\right) & \left(2,6\right) & \left(\frac{5}{2},\frac{5}{2}\right) & \left(4,4\right)\\ iX & \left(6,2\right) & \left(0,0\right) & \left(4,4\right) & \left(\frac{5}{2},\frac{5}{2}\right)\\ U_{3} & \left(\frac{5}{2},\frac{5}{2}\right) & \left(4,4\right) & \left(0,0\right) & \left(6,2\right)\\ U_{4} & \left(4,4\right) & \left(\frac{5}{2},\frac{5}{2}\right) & \left(2,6\right) & \left(5,5\right) \end{array}.$$

**Figure 3:**
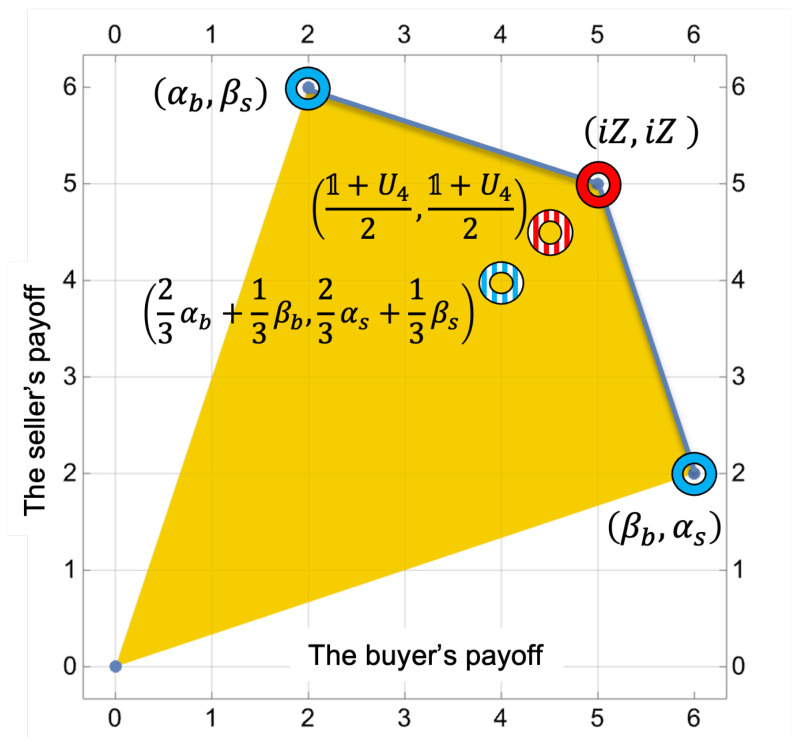
Feasible payoff regions in the Buyer–Seller game: Blue rings mark three classical NE; red rings indicate NE of the quantum game (equilibria in mixed strategies are a striped pattern). Blue line is the Pareto front.

The expansion is generated again by unitary strategies $U_{3}$ and $U_{4}$, defined in (43). The optimal symmetric Nash equilibria are achieved with mixed strategy profiles $\left(\frac{1}{2},0,0,\frac{1}{2}\right)$, $\left(\frac{1}{2},0,0,\frac{1}{2}\right)$, resulting in equal payoffs of $\left(\frac{9}{2},\frac{9}{2}\right)$ for both negotiators; see Figure 3.

The previously mentioned example demonstrates that in the pursuit of quantum frameworks to resolve difficulties in optimizing results within classical games, it is crucial to account for the efficiency and universality of these quantum extensions. Result (45) emerges as the optimal solution, providing an equilibrium for both players, with payoffs maintaining a value of 5, corresponding to mutual honesty, an equilibrium unachievable in the classical context. However, the applicability of this result is limited, as it depends on the sequence in which players execute their strategies. The universal extension (47), which remains invariant with respect to the initial game’s structure, does not achieve complete efficacy. This method has been shown to reduce tendencies towards manipulative low/highball strategies, though it does not entirely eliminate such behaviors. It provides each player with a mixed strategy payoff of the quantum extension $\frac{9}{2}$, which, while suboptimal compared to the ideal payoff of 5, is still better than the payoff of 4 from the best outcome in the classical game.

The quantum formulation of the Buyer-Seller Game demonstrates how entanglement and superposed strategies can reduce the profitability of deception and strengthen the credibility of truthful signaling. Whereas the classical model often sustains equilibria in which misrepresentation is strategically advantageous, the quantum extension enables stable outcomes that reward honesty and support trust between the parties. This finding directly addresses the honesty–deception dilemma, suggesting that quantum strategies can realign incentives in favor of transparency without undermining strategic viability.

## 7. Discussion

Our guiding research question asked whether quantizing negotiation games can produce Nash equilibria that are both strategically stable and consistent with ethical principles such as cooperation, fairness, and honesty. The findings across the three focal dilemmas suggest that this is indeed possible. In the Prisoner’s Dilemma, quantum strategies allow players to escape the mutual-defection trap, making cooperation a stable equilibrium rather than a fragile deviation. In the Ultimatum Game, quantization produces multiple equilibria, including equal surplus divisions—outcomes that are typically unstable or non-credible in the classical form. Likewise, in the Battle of the Sexes, quantum strategies symmetrize payoffs and yield Pareto-optimal equilibria, resolving distributive asymmetries that persist in the classical version. Finally, in the Buyer–Seller Game, entangled strategies eliminate inefficient equilibria such as mutual deception and stabilize truth-telling as the unique equilibrium. Collectively, these cases illustrate how quantum mechanics expands the strategic space and enables players to move beyond the efficiency losses and ethical deadlocks that define classical formulations.

We do not claim that such ethically aligned equilibria arise in all possible quantum extensions. Our analysis is restricted to structurally permissible and symmetry-preserving quantizations that embed the classical game as a special case. Within this constrained and theoretically motivated class, the results demonstrate that ethically desirable equilibria are not pathological artifacts of ad hoc modeling, but robust possibilities enabled by quantum strategic structure.

The broader implication is that quantum strategies allow for efficiency and ethics to reinforce each other rather than remain in conflict. Where classical game theory typically resolves dilemmas in favor of self-interest, quantum models show that fairness, cooperation, and honesty can emerge as normatively desirable and strategically viable. Across the three dilemmas at the heart of negotiation research: cooperation vs. competition, self-interest vs. equity, and honesty vs. deception, quantization consistently shifts equilibria toward outcomes that reconcile rational stability with ethical principles.

This potential comes directly from the mechanics of quantum systems. Entanglement links players’ strategies in ways that mimic implicit coordination, making deception less profitable and cooperation more attractive. Superposition broadens strategic options beyond probabilistic mixtures, enabling nuanced and context-sensitive responses. Together, these features create conditions under which ethical conduct is not a fragile exception but a natural equilibrium of the game. Although the extended games are implemented via quantum mechanisms, the equilibrium analysis is entirely classical in the game-theoretic sense: players face explicit payoff matrices with an enlarged set of strategies and need not understand the underlying physical implementation in order to form rational best responses.

This paper offers several key theoretical contributions to the study of negotiation games and quantum game theory. First, it introduces a novel integration of ethics and strategic rationality by applying quantum game-theoretic tools to negotiation dilemmas such as cooperation, fairness, and honesty. Unlike classical models, which often treat ethical norms as constraints external to the game, our framework embeds them directly into the strategic structure of negotiation analysis.

Second, we demonstrate that quantum equilibria can stabilize ethically desirable behaviors: truth-telling, fair division, and cooperation, which are typically unstable, non-credible, or irrational under classical assumptions. These outcomes emerge not through external enforcement or reputational mechanisms, but through the intrinsic properties of quantum mechanics, particularly entanglement and superposition.

Third, we highlight the methodological significance of quantization choices. Different approaches to defining initial states, entanglement parameters, and measurement protocols can lead to distinct equilibrium structures, emphasizing that the ethical advantages of quantum strategies are contingent on how classical games are formalized in the quantum domain.

Finally, this paper contributes to a reconceptualization of rationality in strategic interaction. By showing that quantum strategies can align ethical norms with payoff optimization, we challenge the conventional dichotomy that pits moral behavior against self-interest. Instead, quantum negotiation games suggest a model in which ethical and rational considerations may be mutually reinforcing rather than mutually exclusive.

The implications of these findings extend far beyond theoretical interest and point toward concrete technological applications. One promising avenue is the design of intelligent negotiation systems involving autonomous agents. With rapid advances in artificial intelligence, large language models, and quantum computing hardware, it is becoming feasible to embed quantum-inspired or even quantum-native strategies into multi-agent systems. Such systems could be designed not only to maximize efficiency but also to adhere to ethical norms such as fairness, transparency, and non-manipulation, criteria increasingly demanded in both public and private governance.

Building on these capabilities, ethical protocols could be implemented in domains such as digital governance, smart contracts, and decentralized resource allocation. For example, blockchain platforms and decentralized autonomous organizations (DAOs) are already experimenting with algorithmic rule enforcement. Quantum negotiation algorithms could enhance these systems by ensuring that contract outcomes satisfy distributive justice while remaining strategically stable. Looking further, the integration of quantum negotiation frameworks into strategically important applications such as climate negotiations, global supply chains, and health resource distribution could help institutions reconcile the dual demands for legitimacy and efficiency. In such contexts, embedding fairness and honesty into the strategic architecture is highly desirable and essential for durable and widely accepted agreements.

Despite these promising theoretical insights, several limitations must be acknowledged. First, the practical implementation of quantum-enhanced negotiation systems remains highly speculative. Although quantum computing has advanced rapidly in recent years, current hardware still faces fundamental challenges of scalability, noise, and error correction. Applications of fully quantum negotiation remain distant, and for the foreseeable future, quantum-inspired algorithms on classical hardware may be more practical than true quantum deployments.

Second, embedding quantum strategies into negotiation systems often requires reliance on complex algorithmic processes that may function as “black boxes.” This raises serious concerns about accountability, explainability, and ethical oversight, issues that are already pressing in AI-driven decision-making. In negotiation contexts, where legitimacy and trust are central, opaque mechanisms could undermine the very ethical goals they are meant to advance.

Finally, this study’s reliance on abstract, stylized game models imposes its own limitations. We assumed rational actors with access to idealized quantum operations, whereas real negotiations are shaped by bounded rationality, cognitive biases, institutional rules, and cultural variation. Our results should therefore be interpreted as a conceptual proof of possibility rather than a ready blueprint for application. Future research should explore how quantum strategies interact with behavioral dynamics, institutional design, and real-world negotiation practices.

The findings of this paper open several promising avenues for future research. A first priority is the development of new equilibrium concepts that account for the ethical and strategic richness of quantum negotiation games, particularly where classical notions fail to capture fairness or implicit coordination through entanglement. Another important direction lies in designing implementable quantum negotiation protocols, especially in distributed AI systems and secure communication networks, as quantum internet infrastructure begins to take shape. Extending the analysis to repeated and multi-party negotiations also offers fertile ground, since quantum effects may sustain long-term cooperation or stabilize coalitions in ways that classical models cannot.

Complementing these theoretical efforts, simulation-based and experimental studies using quantum algorithms could provide insight into how such models perform under realistic conditions shaped by bounded rationality, uncertainty, and noise. Finally, an interdisciplinary research agenda is needed to examine the legal, regulatory, and ethical implications of quantum-enhanced negotiation, ensuring that protocols developed for other applications, e.g., climate policy, international trade, or health resource allocation, are transparent, accountable, and normatively legitimate.

The present work is intended as a conceptual and theoretical contribution; the implementation and empirical testing of quantum negotiation games on quantum hardware or simulators constitute a natural next step and are the subject of our ongoing research.

## 8. Conclusions

This paper aims to examine whether the quantization of negotiation games can enable the emergence of Nash equilibria that are both strategically stable and consistent with ethical principles such as cooperation, fairness, and honesty. Positioned at the intersection of economics, ethics, and quantum theory, our inquiry underscores the persistent tension between strategic rationality and normative ideals in negotiation.

By analyzing the selected negotiation games, we demonstrated that quantum strategies can substantially alter equilibrium outcomes. In contrast to classical games, where equilibria often favor self-interest, their quantum formulations produced solutions that sustained equal division, credible commitments, and truthful signaling, outcomes that are either unstable or strategically irrational in classical frameworks.

The core contribution of this work lies in extending quantum game theory to the study of ethics in negotiation. By evaluating quantum equilibria through both strategic and normative lenses, we show that fairness, cooperation, and honesty need not be excluded from rational equilibrium concepts. Our findings suggest that quantization broadens the range of equilibria considered rational, opening possibilities for solutions that are simultaneously stable and ethically desirable.

Looking ahead, the implications extend beyond theory. As quantum technologies advance and negotiation increasingly involves AI agents, the design of negotiation protocols may integrate quantum mechanisms that embed ethical norms directly into strategic interaction. Rather than treating cooperation, fairness, and honesty as external constraints, quantum games provide conceptual models for institutionalizing them within the very logic of negotiation.

## Figures and Tables

**Table 1:** Prisoner’s Dilemma game payoff matrix.

		Player 2	
		Cooperate	Defect
Player 1	Cooperate	(3, 3)	(0, 5)
	Defect	(5, 0)	(1, 1)

**Table 2:** Battle of the Sexes payoff matrix.

		Bob	
		Ballet (*B*)	Fight (*F*)
Alice	Ballet (*B*)	(3, 2)	(1, 1)
	Fight (*F*)	(0, 0)	(2, 3)

**Table 3:** The buyer–seller game.

		Seller	
		$\alpha_{s}$	$\beta_{s}$
Buyer	$\alpha_{b}$	(5,5)	(2,6)
	$\beta_{b}$	(6,2)	(0,0)

## Data Availability

Data sharing is not applicable to this article, as no new data were generated.
